# Safety Evaluation of *Bifidobacterium breve* IDCC4401 Isolated from Infant Feces for Use as a Commercial Probiotic

**DOI:** 10.4014/jmb.2103.03041

**Published:** 2021-05-24

**Authors:** In Young Choi, Jinhee Kim, Su-Hyeon Kim, O-Hyun Ban, Jungwoo Yang, Mi-Kyung Park

**Affiliations:** 1 School of Food Science and Biotechnology, Kyungpook National University, Daegu 41566, Republic of Korea; 2 Food and Bio-Industry Research Institute, Kyungpook National University, Daegu 41566, Republic of Korea; 3 Drug Information Platform Center, Korea Research Institute of Chemical Technology, Daejeon 34114, Republic of Korea; 4 Ildong BioScience, Pyeongtaek 17957, Republic of Korea

**Keywords:** Safety evaluation, *Bifidobacterium breve*, probiotics

## Abstract

Previously, our research group isolated *Bifidobacterium breve* IDCC4401 from infant feces as a potential probiotic. For this study, we evaluated the safety of *B. breve* IDCC4401 using genomic and phenotypic analyses. Whole genome sequencing was performed to identify genomic characteristics and investigate the potential presence of genes encoding virulence, antibiotic resistance, and mobile genetic elements. Phenotypic analyses including antibiotic susceptibility, enzyme activity, production of biogenic amines (BAs), and proportion of D-/L-lactate were evaluated using E-test, API ZYM test, high-performance liquid chromatography (HPLC), and D-/L-lactic acid assay respectively. The genome of *B. breve* IDCC4401 consists of 2,426,499 bp with a GC content of 58.70% and 2,016 coding regions. Confirmation of the genome as *B. breve* was provided by its 98.93% similarity with *B. breve* DSM20213. Furthermore, *B. breve* IDCC4401 genes encoding virulence and antibiotic resistance were not identified. Although *B. breve* IDCC4401 showed antibiotic resistance against vancomycin, we confirmed that this was an intrinsic feature since the antibiotic resistance gene was not present. *B. breve* IDCC4401 showed leucine arylamidase, cystine arylamidase, α-galactosidase, β-galactosidase, and α-glucosidase activities, whereas it did not show production of harmful enzymes such as β-glucosidase and β-glucuronidase. In addition, *B. breve* IDCC4401 did not produce any tyramine, histamine, putrescine, cadaverine, or 2-phenethylamine, which are frequently detected BAs during fermentation. *B. breve* IDCC4401 produced 95.08% of L-lactate and 4.92% of Dlactate. Therefore, our findings demonstrate the safety of *B. breve* IDCC 4401 as a potential probiotic for use in the food industry.

## Introduction

The United Nations and World Health Organization (WHO) [1] define probiotics as “live microorganisms which confer health benefits on the host when administered in adequate amounts”. Probiotics provide their major health benefits by inhibiting the growth of pathogens in the gastrointestinal tract, reducing the risk of colon cancer and bowel disease, controlling serum cholesterol levels, facilitating digestion, and improving nutrient absorption [2-5]. In addition, they contribute to a balanced gut microbial community and strengthen the immune system. Due to these health benefits and advantages, customer interest in probiotic foods has been increasing. Probiotics have been applied in a wide range of industries such as food, alcoholic beverage, periodontal disease treatment, animal feed and cosmetics [4, 6]. As a result, the global probiotic market size was estimated at approximately 48 billion USD in 2018 and is predicted to reach 77.09 billion USD with a compound annual growth rate of 6.9% by 2025 [7].

Most probiotics are gram-positive, catalase-negative, and non-pathogenic bacteria. There are several bacteria that are used as probiotics such as *Lactobacillus* spp., *Bifidobacterium* spp., *Propionibacterium* spp., *Peptostreptococcus productus*, *Bacillus* spp., *Lactococcus* spp., *Enterococcus faecium*, *Pediococcus* spp., and *Streptococcus* spp.. Of these, *Lactobacillius* spp. and *Bifidobacterium* spp. were the most commonly used as probiotics in the food and pharmaceutical industries [4, 8]. *Bifidobacterium* spp. were first isolated from the feces of breast-fed infants in 1899 [9] and are among the most dominant probiotics during the neonatal period, especially in breast-fed infants [10]. Previous studies have suggested that *Bifidobacterium* spp. are predominantly found in the feces of healthy breast-fed infants contributing to decreased infant diarrhea [11] as well as providing various health benefits as a probiotic [3, 12]. In particular, *Bifidobacterium* spp. play an important role in balancing the gut microbiota such that the food and pharmaceutical industries use *Bifidobacterium* spp. as a starter culture in their products [13]. Therefore, searching new lactic acid bacteria for commercial application is required.

Recently, our research group isolated *B. breve* IDCC4401 from infant feces in Korea. Although *Lactobacillus* and *Lactococcus* are considered as “generally recognized as safe (GRAS),” *Bifidobacterium* spp. require safety evaluation [14]. For example, some probiotics possess genes encoding virulence, antibiotic resistance, and mobile genetic elements, and have deleterious metabolic activities [production of D-lactate, enzyme activity, and biogenic amines (BAs)] [4, 10, 15-17]. Thus, an FAO/WHO Expert Consultation has recognized and emphasized the necessity for systematic evaluation guidelines for probiotics prior to their commercialization [18]. Following these guidelines for probiotics in food, the safety of isolated novel strain of *B. breve* IDCC4401 was evaluated through genomic analysis to determine the presence of virulence genes, antibiotic resistance genes, and mobile genetic elements. In addition, *B. breve* IDCC4401 was examined for antibiotic susceptibility, enzyme activity, production of BAs, and the proportion of D-/L-lactate formed during incubation.

## Materials and Methods

### Bacterial Culture and Growth Conditions

*B. breve* IDCC4401 obtained from Ildong BioScience Co. (Korea) and grown in 15 ml of MRS broth (Difco Laboratories Inc., USA) at 37°C for 24 h with 0.5% CO_2_ in a static incubator. After centrifugation at 6,000 ×*g* for 15 min, the pellet was resuspended in PBS (pH 7.4; Life Technologies Ltd., UK) and its concentration was adjusted to 10^8^ CFU/ml and 10^9^ CFU/ml by measuring the optical density at 640 nm.

### Whole Genome Sequencing of *B. breve* IDCC4401

Genomic DNA of *B. breve* IDCC4401 was extracted using a Maxwell 16 LEV Blood DNA Kit and a Maxwell 16 Buccal Swab LEV DNA Purification Kit (Promega Co., USA) according to the manufacturer’s instructions. Genome sequencing was performed by Macrogen Inc. (Korea) using a PacBio RS II instrument (Pacific Biosciences of California Inc., USA) with an Illumina platform (Illumina Inc.,USA). Raw data were assembled using the Hierarchical Genome Assembly Process and the assembled gene was predicted and annotated using Prokka v1.13. For additional annotation, predicted protein sets were then analyzed using InterProScan v5.30-69.0 and psiblast v2.4.0 with EggNOG DB v4.5. Average nucleotide identity (ANI) was calculated using average nucleotide identity.py script of PYANI v0.2.10 with ANIm option. To confirm the presence of antibiotic resistance genes and virulence factors, all identified coding sequences (CDS) were compared against the Comprehensive Antibiotic Resistance Database (CARD) [19] and Virulence Factor Database (VFDB) [20], respectively. Prophage regions were identified using the PHASTER web-based program [21]. Transposases were annotated using BLASTP and transposases and conjugal transfer proteins were retrieved from the NCBI GenBank. Circular maps depicting each contig were generated using Circos v0.69.3. The complete genome sequence of *B. breve* IDCC4401 was made available in the GenBank database under nucleotide sequence accession number KP325411.

### Antibiotic Susceptibility of *B. breve* IDCC4401

The antibiotic susceptibility of *B. breve* IDCC4401 was determined by the E-test method against 9 antibiotics according to the European Food Safety Authority (EFSA) guidelines [17]. An overnight culture of bacteria (10^8^ CFU/ml) was swabbed onto 15-cm diameter MRS agar plates with a sterilized cotton swab prior to placing an E-test strip (Liofilchem Inc., Italy) on the surface of the plate. After incubation at 37°C for 18 h, the relevant inhibition ellipse intersected the strip and the minimum inhibitory concentration was determined at complete inhibition. Finally, antibiotic susceptibility of *B. breve* IDCC4401 was determined following the guidelines of the EFSA [17].

### Enzyme Activities of *B. breve* IDCC4401

The enzyme activities of *B. breve* IDCC4401 were determined using the API ZYM Kit (Biomerieux Inc., France) according to the manufacturer’s instructions. The overnight culture of *B. breve* IDCC4401 (109 CFU/ml) was added into a cupule containing different substrate solutions and incubated at 37°C for 4 h. One drop of ZYM A and ZYM B reagents was added sequentially prior to incubation for 5 min at RT. Color change of the mixture was graded from zero (no activity) to five by comparing color intensity with the color chart provided by the manufacturer. Positive enzyme activities were determined to be above three intensity levels of color change.

### BA Production of *B. breve* IDCC4401

Production of BAs by *B. breve* IDCC4401 was investigated following the method described in a previous study [22] with minor modifications. The overnight cultured *B. breve* IDCC4401 was centrifuged at 2,300 ×*g* for 5 min at 4°C. An aliquot of 0.75 ml of supernatant was mixed with the same volume of 0.1 M HCl and filtered through a 0.45-μm membrane to extract BAs. For derivatization of the BAs, 1 ml of filtered BAs was incubated at 70°C for 10 min prior to addition of 200 μl of saturated NaHCO_3_, 20 μl of 2 M NaOH, and 0.5 ml of dansyl chloride solution (10 mg/ml of acetone). The derivatized BAs were mixed with 200 μl of proline (100 mg/ml of H_2_O) and incubated for 15 min at RT in the dark. The derivatized BAs were then separated and quantified using HPLC (LC-NETII/ADC, JASCO Inc., Japan) with an Athena C18 column (4.6 mm × 250 mm, ANPEL Laboratory Technologies Inc., China). Aqueous acetonitrile solution (Sigma-Aldrich Co., USA) was used as a mobile phase and the flow rate was adjusted to 0.8 ml/min. Finally, a peak was detected at 254 nm using a UV detector (UV-2075 Plus, JASCO Inc., Japan). The detected BAs were quantified from calibration curves of BAs including tyramine, histamine, putrescine, 2-phenethylamine, and cadaverine (Sigma-Aldrich Co.).

### Proportion of D-/L-Lactate of *B. breve* IDCC4401

An overnight culture of *B. breve* IDCC4401 was centrifuged at 2,300 ×*g* for 30 min at 4°C and the supernatant was collected. Following supernatant filtration using a 0.2-μm pore size membrane, the filtrate was mixed with the agents in a D-/L-Lactic Acid (D-/L-Lactate) (Rapid) Assay Kit (Megazyme, Ireland). Absorbances of the mixture were measured at 340 nm and the concentration of D-/L-lactate was calculated according to the manufacturer’s protocol.

## Results and Discussion

### Whole Genome Sequencing of *B. breve* IDCC4401

**Fig. 1:**
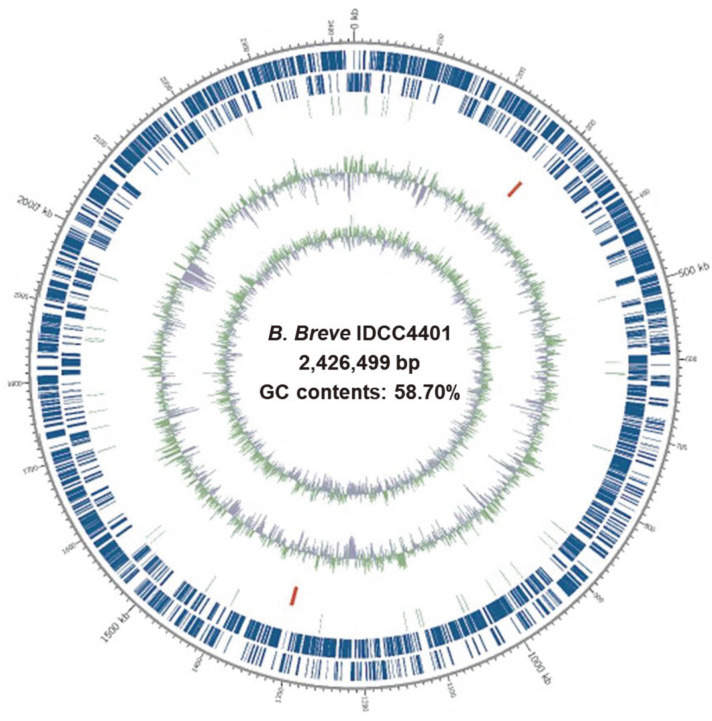
Genomic map of *B. breve* IDCC4401. Marked genome characteristics are shown from outside to the center; CDS on forward strand, CDS on reverse strand, tRNA, rRNA, GC content and GC skew.

Whole genome sequencing of *B. breve* IDCC4401 was performed for the identification and confirmation of genes encoding antibiotic resistance, virulence, and mobile genetic elements to ensure safety. The assembled genome consisted of 2,426,499 bp with a GC content of 58.70% (Fig. 1). The strain was confirmed as *B. breve* with a similarity of 98.93% with *B. breve* DSM20213 based on ANI analysis. Of a total of 2,016 CDSs of *B. breve* IDCC4401, 1,583 CDSs were annotated as functional genes involved in translation, ribosomal structure, biogenesis, RNA processing, modification, transcription, replication, recombination, repair, cell cycle control, cell division, chromosome partitioning, defense mechanisms, signal transduction mechanisms, and 433 unknown genes (Table 1). Based on CARD and VFDB, genes associated with virulence and antibiotic resistance were not found in *B. breve* IDCC4401, respectively. Although 33 transposases, as mobile genetic elements, were identified, these mobile elements were not involved in the acquisition and transfer of antibiotic resistance genes due to the absence of virulence and antibiotic resistance genes in *B. breve* IDCC4401. Therefore, this result confirmed the safety of *B. breve* IDCC4401 for use as a probiotic based on our thorough genome analysis.

### Antibiotic Susceptibility (MICs) of *B. breve* IDCC4401

To ensure safety, the phenotypic antibiotic susceptibility of *B. breve* IDCC4401 was investigated against 9 antibiotics including ampicillin (one representative of a β-lactam antibiotic), gentamicin, streptomycin, erythromycin, clindamycin, tetracycline, chloramphenicol, vancomycin, and kanamycin, using the E-test method [18]. Determination of resistance and susceptibility against each antibiotic followed the EFSA guidelines. As shown in Table 2, *B. breve* IDCC4401 was susceptible to ampicillin, gentamicin, streptomycin, erythromycin, clindamycin, tetracycline, and chloramphenicol but resistant to vancomycin. Susceptibility to kanamycin could not be determined because EFSA did not provide a definite cut-off value. However, the MIC value (256 μg/ml) against kanamycin was 2 times lower than that of *B. longum* BORI (512 μg/ml), and 4 times lower than those of *B. longum* BB536, *B. breve* M-16, *B. bifidum* BGN4, and *B. lactis* BB-12 (1,024 μg/ml) that were considered as GRAS [16].

Charteris *et al*. (1999) investigated the antibiotic susceptibilities of two *B. breve* strains (15698 and 15701) isolated from human gastrointestinal tract and found resistances against five β-lactam antibiotics (penicillin G, amoxycillin, cepharadine, ceftizoxime and cefotaxime) among 44 antibiotics considered as “last resort antibiotics” [23]. In the meantime, *B. breve* IDCC4401 exhibited susceptibility to ampicillin. In addition, two other *B. breve* strains (IF2-173 and IF2-174) also isolated from breast-fed infants were susceptible to ampicillin, streptomycin, and chloramphenicol whereas they were resistant to tetracycline, erythromycin, and vancomycin [10]. When compared with previous study [10], *B. breve* IDCC4401 showed narrow antibiotic resistance only to vancomycin. Although *B. breve* IDCC4401 showed antibiotic resistance against vancomycin, this result could be related to an intrinsic feature of *B. breve* IDCC4401 as vancomycin resistance is a general feature of most *Bifidobacterium* spp. as shown in a study by Charteris *et al*. [23]. Vancomycin resistance is thought to be a result of the presence of D-alanine residues in the cell wall preventing vancomycin binding [24, 25].

### Enzyme Activity of *B. breve* IDCC4401

The enzyme activity of *B. breve* IDCC4401 was investigated against 19 different enzymes using the API ZYM assay, which involved degradation of peptides, phosphomonoesters, lipids, mucopolysaccharides, polysaccharides, chitin, cellulose, starch, and galactan (Table 3) [26]. For example, leucine and cysteine arylamidases are involved in the degradation of peptides whereas α-galactosidase, β-galactosidase, and α-glucosidase participate in the degradation of carbohydrates. *B. breve* IDCC4401 exhibited leucine arylamidase, cystine arylamidase, α-galactosidase, β-galactosidase, and α-glucosidase activities among the 19 different enzymes.

Desjardins *et al*.’s study demonstrated that four *B. breve* strains (ATCC 15698, ATCC 15699, ATCC 15700, and ATCC 15701) obtained from the American Type of Culture Collection (ATCC) exhibited the activities of esterase lipase, leucine aminopeptidase, acid phosphatase, phosphoamidase, α-galactosidase, β-galactosidase, α-glucosidase, and β-glucosidase [27]. In addition, *B. breve* ATCC 15699 showed β-glucuronidase activity. The enzyme activities of α-galactosidase, β-galactosidase and α-glucosidase were found to be consistent with those of a previous study [27]. Chevalier *et al*. (1990) demonstrated that the presence of *Bifidobacterium* spp. in the feces of the healthy infants was accompanied by α-galactosidase and α-glucosidase, which are characteristics of *Bifidobacterium* spp.[28]. Further, evidence proved that α-galactosidase and β-galactosidase enzyme activities were highly produced in *B. longum* RD 47, suggesting that these enzymes are involved in hydrolyzing galactosides such as lactose [29]. These findings are consistent with our results obtained from this study. Although probiotics can improve digestion and probiotic enzymes can act as natural substances for digestion of food in the human body, some enzyme activities may produce compounds that are to the host [30]. Cole and Fuller (1986) reported that β-glucosidase may produce aglycones which are linked to the development of colorectal cancer [31]. Moreover, β-glucuronidase might be linked to carcinogenic compounds for colorectal cancer [32]. Since *B. breve* IDCC4401 did not show the activity of β-glucosidase and β-glucuronidase, it appears to be safe and suitable for use as a probiotic.

### BA Production of *B. breve* IDCC4401

Lactic acid bacteria (LAB) including *Bifidobacterium* spp. and *Lactobacillus* spp. can produce BAs during the fermentation process [33]. Previous studies [4, 34] reported that tyramine, histamine, putrescine, cadaverine, 2-phenethylamine, and spermidine can be frequently detected in fermented foods. The problem is that these BAs, at high concentration, can cause toxicological effects to humans with certain symptoms such as respiratory distress, heart palpitation, hypertension or hypotension, headaches, and allergenic disorders [33]. Verifying the formation of BAs by *B. breve* IDCC4401 is required to ensure the safety of *B. breve* IDCC4401. As shown in Table 4, *B. breve* IDCC4401 did not produce any of the five representative BAs, indicating that it did not produce any harmful BAs.

According to Lorencova *et al*.’s study [35], five *Bifidobacterium* strains (*Bifidobacterium* spp., *B. adolescentis*, *B. lactis*, *B. bifidum*, and *B. longum*) produced tyramine, cadaverine, putrescine, and spermidine among the eight BAs including the five we tested and three others (tryptamine, speramine, and spermidine). However, Kim *et al*.’s study [16] exhibited putrescine formation by *B. bifidum* BGN4 and *B. longum* BORI, but not cadaverine, histamine, or tyramine formation. Contradictory to previous studies, Ku *et al*. [4] demonstrated that *B. lactis* AD011 did not form any BAs when tested for cadaverine, histamine, putrescine, and tyramine formation, in accordance with our results. Thus, the absence of BAs suggested the potential of *B. breve* IDCC4401 as a commercial probiotic.

### Proportion of D-/L-Lactate of *B. breve* IDCC4401

Lactate produced in either D-form or L-form isomers during fermentation by LAB and exhibits distinct biological effects in human [36]. However, *Lactobacillus* and *Bifidobacterium* have been known as D-lactate producers [37, 38]. Unlike L-lactate, excess D-lactate produced by LAB cause short bowel syndrome, chronic fatigue and metabolic disorders, especially when jejunoileal bypass surgery is performed [39]. Due to these toxicological effects, the proportion of D-/L-lactate of *B. breve* IDCC4401 needs to be clarified for its safety evaluation. As shown in Table 5, *B. breve* IDCC4401 produced 95.08% (21.26 g/l) of L-lactate and 4.92% (0.93 g/l) of D-lactate.

Since the guidelines provided by FAO/WHO did not provide any clear criterion for the ratio of L-lactate and D-lactate, these results were compared with other studies recognized as GRAS. According to Munoz *et al*.’s study [40], *B. longum* CECT 7210 produced 2.22% (0.06 g/l) of D-lactate, which was lower than the L-lactate (97.78%, 2.64 g/l). Although the amount and ratio of D-lactate produced by *B. breve* IDCC4401 was higher than that of *B. longum* CECT 7210, it was lower than that of L-lactate and this pattern was similar to a previous study. In the case of *B. lactis* BB-12^®^ which was certified as GRAS by the FDA, the ratio of L-lactate was more than 95% [41]. In this aspect, it can be assumed that *B. breve* IDCC4401 poses no safety concern over this property.

In conclusion, since the safety of *B. breve* IDCC4401 isolated from infant feces has not yet been investigated for its commercial usage as a probiotic, we investigated the presence of genes encoding virulence, antibiotic resistance, mobile genetic elements, and deleterious metabolic activities in this strain to ascertain its safety as a probiotic. Although *B. breve* IDCC4401 was evaluated for safety in this study, further studies are required on functional characteristics such as resistance to gastric acid and bile salts, adherence ability (aggregation properties and cell hydrophobicity), and antimicrobial activity to ensure its safety as a commercial probiotic in the food industry.

## Figures and Tables

**Table 1:** Annotated functional genes in *B. breve* IDCC4401.

Function	Number of CDS	Ratio (%) of CDS
Translation, ribosomal structure, and biogenesis	134	6.6468
RNA processing and modification	1	0.0496
Transcription	125	6.2004
Replication, recombination, and repair	191	9.4742
Cell cycle control, cell division, chromosome partitioning	19	0.9425
Defense mechanisms	52	2.5794
Signal transduction mechanisms	53	2.6290
Cell wall/membrane/envelope biogenesis	90	4.4643
Intracellular trafficking, secretion, and vesicular transport	14	0.6944
Posttranslational modification, protein turnover, chaperones	51	2.5298
Energy production and conversion	47	2.3313
Carbohydrate transport and metabolism	231	11.4583
Amino acid transport and metabolism	166	8.2341
Nucleotide transport and metabolism	62	3.0754
Coenzyme transport and metabolism	34	1.6865
Lipid transport and metabolism	28	1.3889
Inorganic ion transport and metabolism	95	4.7123
Secondary metabolites biosynthesis, transport and catabolism	9	0.4464
General function prediction only	181	8.9782
Unknown function	433	21.4782

**Table 2:** Minimal inhibitory concentration (MIC) and antibiotic susceptibility of *B. breve* IDCC4401.

	Cut-off value (µg/ml)	MIC (µg/ml)	Assessment
Ampicillin	2	0.25–0.5	S^1^
Vancomycin	2	>512	R^2^
Gentamycin	64	32	S
Kanamycin	–^3^	256	–
Streptomycin	128	64–128	S
Erythromycin	1	0.125–0.25	S
Clindamycin	1	0.25	S
Tetracycline	8	2	S
Chloramphenicol	4	4	S

^1^S, Susceptible

^2^R, Resistant

^3^-, Cut-off value is not established in EFSA guidelines.

**Table 3:** Enzyme activities of *B. breve* IDCC4401 determined by API ZYM test.

Enzyme	Activity^*^
Alkaline phosphatase	−
Esterase	−
Esterase Lipase	−
Lipase	−
Acid phosphatase	−
Naphthol-AS-BI-phosphohydrolase	−
Leucine arylamidase	+
Valine arylamidase	−
Cystine arylamidase	+
Trypsin	−
α-chymotrypsin	−
α-galactosidase	+
β-galactosidase	+
β-glucuronidase	−
α-glucosidase	+
β-glucosidase	−
N-acetyl- β-glucosaminidase	−
α-mannosidase	−
α-fucosidase	−

*+, enzyme activity; –, no enzyme activity

**Table 4:** Biogenic amine production of *B. breve* IDCC4401.

Biogenic amine (µg/ml)				
Tyramine	Histamine	Putrescine	Cadaverine	2-Phenethylamine

ND.	ND.	ND.	ND.	ND.

ND: not detected.

**Table 5:** L-/D-lactate production of *B. breve* IDCC4401.

L-lactate (g/L)	D-lactate (g/L)	L-form (%)	D-form (%)
21.26	0.93	95.08	4.92

The data represent the mean ± SD (*n* = 3).
